# An Analysis of Dentigerous Cysts Developed around a Mandibular Third Molar by Panoramic Radiographs

**DOI:** 10.3390/dj7010013

**Published:** 2019-02-04

**Authors:** Masahiko Terauchi, Satoshi Akiya, Junya Kumagai, Yoshio Ohyama, Satoshi Yamaguchi

**Affiliations:** 1 Department of Maxillofacial Surgery, Graduate School of Medical and Dental Sciences, Tokyo Medical and Dental University, 1-5-45 Yushima, Bunkyo-ku, Tokyo 113-8549, Japan; teraorg@tmd.ac.jp (M.T.); junya0722@hotmail.com (J.K.); yohyama1219@gmail.com (Y.O.); 2 Center for advanced dental clinical education of Dental Hospital, Tokyo Medical and Dental University, 1-5-45 Yushima, Bunkyo-ku, Tokyo 113-8549, Japan; satoshiakiya3313@gmail.com; 3 Department of Dentistry, Tokyo Yamate Medical Center, 3-22-1 Hyakunin-cho, Shinjuku-ku, Tokyo 169-0073, Japan; 4 Department of Oral and Maxillofacial Surgery, Shizuoka City Hospital, 10-93 pursuer-cho, Aoi-ku, Shizuoka City, Shizuoka 420-8630, Japan

**Keywords:** dentigerous cyst, mandibular third molar, panoramic radiograph

## Abstract

Dentigerous cysts are one of the most prevalent types of odontogenic cysts and are associated with the crown of an unerupted tooth, especially of the mandibular third molar. In this study, the characteristics of a dentigerous cyst developed around a mandibular third molar on panoramic radiographs were investigated. The panoramic images of 257 consecutive dentigerous cyst cases associated with a mandibular third molar were analyzed. The mean age of the patients was 45.9 ± 13.3 years. The size of the cyst did not significantly correlate to the age of the patient. The unilocular type (89.1%) and the crown side type (68.5%) were significant. The associated mandibular third molars had a high frequency of class III (64.6%) and position B (48.3%) in Pell and Gregory classification and of horizontal position (36.3%) in angulation. Dentigerous cysts were thought to originate and grow commonly around deeply impacted third molars. The associated third molar with dentigerous cyst tends to have a mesial inclination. Dentigerous cysts do not appear to develop gradually after the crown formation has finished, but arise at various periods randomly.

## 1. Introduction

The jawbones have a high prevalence of cysts in the human body due to the abundant amount of epithelial remnants. Dentists, therefore, frequently encounter cystic lesions within jawbones. Most jaw cysts are lined with epithelium derived from the odontogenic epithelium. These are referred to as odontogenic cysts. They are subclassified into two groups, developmental or inflammatory [1]. Dentigerous cysts are the second most common odontogenic cyst and the most common developmental odontogenic cyst. A previous systematic review regarding odntogenic cysts showed that of 18,297 odontogenic cysts, 9982 (54.6%) were cases of radicular cysts, and 3772 (20.6%) were cases of dentigerous cysts [2].

Dentigerous cysts are usually asymptomatic and discovered accidentally as radiolucencies on panoramic radiographs taken for general dental treatment or from investigations of the reason for delayed tooth eruption. The panoramic radiograph, therefore, is the first indication of dentigerous cysts in many cases. It is important for the dentist to understand the characteristics of the dentigerous cyst obtained from a panoramic radiograph. This cyst is associated with the crown of an impacted, embedded, or unerupted permanent tooth. The most frequently involved tooth is a mandibular third molar [3,4].

In this study, we examined the characteristics of 257 dentigerous cysts developed around a mandibular third molar on panoramic radiographs. The shape, position and size of the radiolucent area showing the cyst region were surveyed. It is well known that radiographically, a dentigerous cyst typically presents a unilocular radiolucency with a corticated margin and its radiolucent area surrounds the crown of the associated impacted tooth [1]. This typical type is not the only thing and there are other types. The concrete frequencies of those types were examined because there was no information about its frequencies in previous reports. New evaluation approaches regarding the size of cyst were introduced to reduce the individual differences. The relationship between the size of the cyst and the patient’s age was also analyzed. In addition, the position and angulation of the associated mandibular third molars, which have been hardly reported up to now, were also investigated.

## 2. Materials and Methods

### 2.1. Subjects

The study included 257 consecutive patients who underwent the enucleation of a dentigerous cyst and extraction of the associated mandibular third molar at the dental hospital of Tokyo Medical and Dental University between 2009 and 2013. The diagnosis of all dentigerous cysts was provided pathologically.

All procedures performed in studies involving human participants were in accordance with the ethical standards of the institutional and/or national research committee and with the 1964 Helsinki declaration and its later amendments or comparable ethical standards. The present study was approved by the Institutional Review Board of the Faculty of Dentistry, Tokyo Medical and Dental University (No.1247).

### 2.2. Images

All digital panoramic radiographs were taken using a Super Veraviewepocs (Morita Corp., Kyoto, Japan) instrument operated at 79–80 kVp and 9–10 mA with a photostimulated phosphor plate (ST-IV; Fuji Film Medical Co., Ltd., Tokyo, Japan). The plates were processed with an FCR 7000 system (Fuji Film Medical Co., Ltd., Tokyo, Japan), and the images were stored in the DICOM format. This apparatus provides panoramic images with uniform magnification in both vertical and horizontal dimensions.

### 2.3. The Site, Shape and Position of the Cyst

**Figure 1:**
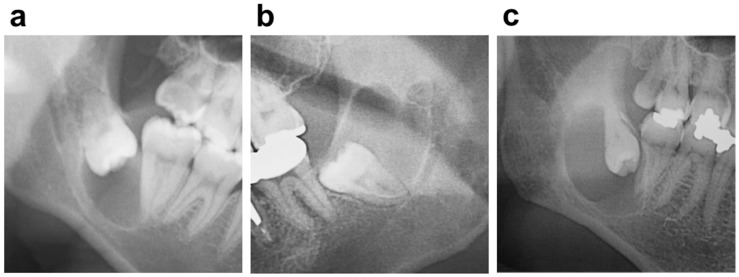
A dentigerous cyst (**a**) unilocular and crown side type; (**b**) multilocular type; (**c**) whole-tooth type.

The Clinical features based on the radiolucent area on panoramic radiographs included the site, locular type, and position of the cyst. The locular types were divided into a unilocular type (Figure 1a) and a multilocular type (Figure 1b). The positions of the cysts were divided into a crown side type (the cyst surrounds the crown of the associated third molar) (Figure 1a) and a whole-tooth type (the cyst surrounds the crown and root of the associated third molar) (Figure 1c).

### 2.4. The Size of the Dentigerous Cyst

The size of the dentigerous cyst or the radiolucent area on panoramic radiograph was examined. We introduced a relative cyst size for a maximum width of the tooth crown. The size of the cyst was evaluated as follows to reduce the individual differences.

The relative size of dentigerous cyst = the length of the major axis of the radiolucent area/the maximum width of the tooth crown of the associated third molar.

### 2.5. The Position and Angulation of the Associated Third Molar

The position of the associated third molar was classified by Pell and Gregory classification [5] and described as follows [6].

(Relation of the tooth to the ramus of the mandible and second molar)

Class I: Sufficient amount of space exists for the accommodation of the mesiodistal diameter of the crown of the third molar.

Class II: The space between the ramus and distal side of the second molar is less than the mesiodistal diameter of the third molar.

Class III: All or most of the third molar is located within the ramus. 

(Relative depth of the third molar in the bone)

Position A: The highest portion of the tooth is on a level with or above the occlusal line.

Position B: The highest portion of the tooth is below the occlusal plane but above the cervical line of the second molar. 

Position C: The highest portion of the tooth is below the cervical line of the second molar tooth in relation to the long axis of the impacted second molar. 

(Angulation)

The angulation of the associated third molar was determined by the method of Quek et al. [7]. A group of cases with “other, 111° to −80°,” in the previous report were classified as Inversion in this study. In a survey of the position and angulation of associated third molars, the cases (n = 17) in which the adjacent second molar was lost were excluded.

### 2.6. Statistics

Fisher’s exact probability was used for statistical analyses. A *p*-value of less than 0.05 was considered significantly different. The values are expressed as mean ± standard deviation (SD). 

## 3. Results

### 3.1. Subjects

**Figure 2:**
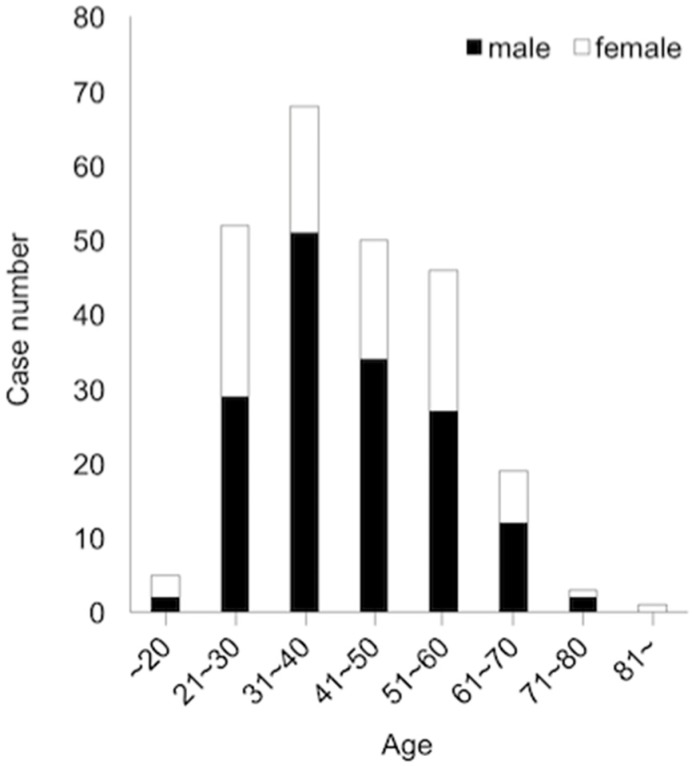
The age and gender distribution of 257 patients with dentigerous cysts developed around a mandibular third molar.

This series included 167 males (65%) and 90 females (35%), a 1.86:1 male/female ratio. Age and gender distribution are shown in Figure 2. Although the highest frequency of dentigerous cysts was present in patients in their 30s, the mean age of our patients at the first visit was 46.0 ± 13.4 years, with a range from 19 to 89 years (Figure 2). 

### 3.2. The Site, Shape and Position of the Cyst

There was no bilateral difference in the mandible (left, 52.1%; right, 47.9%). The unilocular (89.1%) in the locular type and the crown side type (68.5%) in the position of the cyst were significant (*p*-value < 0.05), respectively (Figure 1, Table 1). 

### 3.3. The Size of Dentigerous Cyst

**Figure 3:**
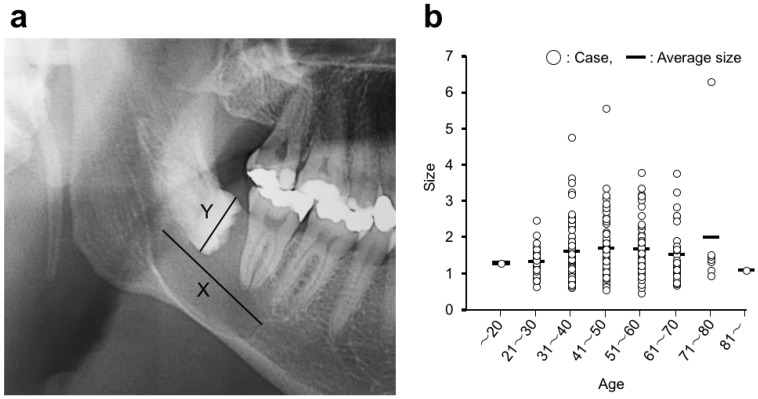
(**a**) The evaluation of the dentigerous cyst size on a panoramic radiograph. Cyst size = Length of the major axis of the radiolucent area (X)/Maximum width of tooth crown of associated third molar (Y). (**b**) Distribution of dentigerous cyst sizes.

We introduced a new method measuring a relative cyst size for a maximum width of the tooth crown (Figure 3a). The average length of the major axis of the radiolucent area and the width of the tooth crown were 22.80 mm and 14.35 mm, respectively. The distribution of a relative cyst size according to age bracket is shown in Figure 3b. The average of a relative cyst size in each age bracket was ranged from 1 to 2. There was no significant difference in the relative size from each age bracket.

### 3.4. The Position and Angulation of the Associated Mandibular Third Molar 

Next, we examined the position of the associated mandibular third molars with the dentigerous cyst, by using Pell and Gregory classification (Table 2). In the horizontal classification, which is based on the amount of tooth covered by the anterior border of the ramus, Class III (64.6%) was the highest frequency. In the vertical classification, Position B (48.3%) was the highest frequency, followed by Position A. The angulation of the associated third molars was determined by the angle formed between the intersected longitudinal axes of the second and third molars (Table 3). The horizontal position showed the highest frequency (36.3%), followed by inversion (32.5%) and mesio-angular (25.4%) angles. The angle of inversion type was +100°~+140° in most cases. 

## 4. Discussion

Patients with dentigerous cysts had a mean age of 46.0 ± 13.4 years in this study, although the highest frequency of dentigerous cysts was present in patients in their 30s. The reason for this is that the frequency of patients in their 40s or older is relatively high compared with that of those in their 20s or younger. There were two types of previous reports on the age distribution of dentigerous cyst.

One showed the mean age of the patients as those in their 30s [3,4], the other showed that it was patients in their 40s [8,9,10]. It is difficult to understand this difference in age distribution. The extraction of the impacted tooth, especially of an impacted third molar in a relatively young patient, may be involved [4]. Regarding gender distribution, the previous reports including this study showed a slight male predilection [3,4,10], and an obvious male predilection was indicated in other reports [8,9,11]. 

The site, locular type, and position of the dentigerous cysts in this study are the same as that which is commonly accepted. The unilocular type (89.1%) and crown side position (68.5%) were significantly different. However, the frequencies of the multilocular type (10.9%) and whole tooth position (31.5%), especially the whole tooth position, were higher than our expectations, although it could not be compared with the other data for lack of a previous study. Some large dentigerous cysts have a multilocular image on panoramic radiographs, thought to be due to the persistence of bone trabeculae within the radiolucency, and there are no truly multilocular dentigerous cysts [1]. In fact, dentigerous cysts are largely and pathologically unilocular processes. Dentigerous cysts that give the impression of a multilocular process, however, need to be clinically distinguished from odontogenic tumors or cysts such as ameloblastoma or an odontogenic keratocysts [8,11].

New evaluation approaches for the size of dentigerous cysts were introduced to reduce the individual differences in this study. In this new approach, the image distortion in panoramic radiograph was not considered. There, therefore, were some differences caused by the image distortion among each measurement although all digital panoramic radiographs were taken using the same apparatus under the same photographing condition [12]. A dentigerous cyst originates from the reduced enamel epithelium between the follicle and the tooth crown and develops after the crown of the tooth has been completely formed [10]. We, therefore, thought that the size of a dentigerous cyst became larger with age at the outset. Actually, however, there was no significant correlation between the size and age. In this relationship, the same result as ours was reported in two previous papers, even though their measurement methods differed with the one we used in this study [11,13]. To the best of our knowledge, no report showed the significant correlation between the size of a dentigerous cyst and the age of the patient. Dentigerous cysts do not appear to develop gradually after the crown formation has finished, but arise at various periods randomly. The size measured in this study is two-dimensional of the radiolucent area on a panoramic radiograph, and there is no information about buccolingual size. The relationship between three-dimensional or buccolingual size and age is uncertain. A dentigerous cyst is capable of achieving a significant size, occasionally with a painless expansion of cortical bone in the involved area, but a large size that causes pathologic fracture is rare [14]. This behavior of dentigerous cyst is different from ameloblastoma or odontogenic keratocysts [11,15,16]. 

The position of the associated third molars with dentigerous cysts was examined by Pell and Gregory classification. No previous papers examined the position of the associated third molars with dentigerous cysts by using this classification, as far as we knew. In horizontal classification, class III was the highest frequency, followed by classes II and I. In other words, third molars whose crowns are fully covered by bone have the highest frequency, and the frequency increases with the amount of tooth covered by the ascending mandibular ramus. Dentigerous cysts originate from the reduced enamel epithelium and grow by internal pressure generated by fluid drawn into the space between the reduced enamel epithelium and the tooth crown [1,17]. Considering that, it is easy to understand that dentigerous cysts grow easily around the tooth whose crown is fully surrounded by bone because internal pressure by fluid appears more easily and rises in hard tissue more so than in soft tissue. This is why the amount of tooth covered by the mandibular ramus is related to the frequency of dentigerous cysts.

In vertical classification, Class B (48.3%) had the highest frequency, followed by Class A (32.1%) and Class C (19.6%). We previously examined the position of the mandibular third molars of 1906 extraction cases that do not have a dentigerous cyst or other pathologies. This result showed that Class A (85.1%) had the highest frequency, followed by Class B (12.9%) and Class C (1.9%) (data not shown). When we think of these two data instances together, dentigerous cysts are thought to originate and grow commonly around deeply impacted third molars.

The angulation of an associated third molar was determined by the angle formed between the intersected longitudinal axes of the second and third molars. There has been no previous report on this angulation as with the position of the associated third molar. The horizontal position had the highest frequency (36.3%), followed by inversion (32.5%) and mesio-angular positions (25.4%). This result indicates clearly that the third molar associated with a dentigerous cyst tends to have a mesial inclination. In the original report, which proposed the angular classification of mandibular third molar used in this study, the mesio-angular position (59.5%) is the most common, followed by the horizontal (17.6%), disto-angular (9.8%), and vertical (9.5%) positions [7]. The horizontal and mesio-angular positions rank higher in both results, even though the order is reversed. The greatest difference between the two results is the frequency of inversion. It is second ranked (30.5%) in this study but low (0.6%) in the examination of mandibular third molars that do not have a dentigerous cyst. There is a possibility that the inverted position of mandibular third molars associated with a dentigerous cyst is a consequence of tooth realignment by cyst enlargement.

## 5. Conclusions

The patients with dentigerous cyst had a mean age of 46.0 ± 13.4 years in this study, although the highest frequency of dentigerous cyst was present in patients in their 30s. The panoramic images of dentigerous cyst developed around a mandibular third molar showed some diagnostics; (1) a unilocular radiolucent area surrounded the crown of associated third molar; (2) the size of the cyst was not related to the age of a patient; (3) the associated mandibular third molars were deeply impacted and had a mesial inclination. 

These findings lead to the following characteristics of a dentigerous cyst. Dentigerous cysts were thought to originate and grow commonly around deeply impacted third molars. Dentigerous cysts do not appear to develop gradually after the crown formation has finished, but arises at various periods randomly.

## Figures and Tables

**Table 1:** The clinical features of dentigerous cysts on panoramic radiographs.

Clinical Feature	Frequency	Percentage
Site		
Left	134	52.1%
Right	123	47.9%
Locular Type		
Unilocular	229	89.1% *
Multilocular	28	10.9%
Position of the Cyst		
Crown Side	176	68.5% *
Whole Tooth	81	31.5%

* *p*-value < 0.05

**Table 2:** Pell and Gregory classification of the associated mandibular third molars.

Position/Class	I	II	III	Total
A	14 (5.8%)	17 (7.1%)	46 (19.2%)	77 (32.1%)
B	9 (3.8%)	34 (14.2%)	73 (30.4%)	116 (48.3%)
C	7 (2.9%)	4 (1.7%)	36 (15.0%)	47 (19.6%)
Total	30 (12.5%)	55 (22.9%)	155 (64.6%)	240 (100.0%)

Class I, II, III: Horizontal classification (Relation of the tooth to the ramus of the mandible and second molar); Position A, B, C: Vertical classification (Relative depth of the third molar in the bone).

**Table 3:** The associated mandibular third molar angulation.

Angulation Type	The Relationship between the Second and Third Molar	Angle (°)	Frequency	Percentage
Vertical	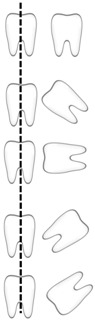	−10~+10	11	4.5%
Mesioangular		+10~+80	61	25.4%
Horizontal		+80~+100	87	36.3%
Distoangular		−10~−80	3	1.3%
Inversion		−80~+100	78	32.5%
Total	-	-	240	100.0%
